# Comparison of the BluePoint MoldID oligonucleotide array and Bruker Biotyper MALDI-TOF MS for the identification of filamentous fungi

**DOI:** 10.1128/jcm.01048-24

**Published:** 2024-12-05

**Authors:** Chia-Hua Chou, Qiao-Ting Chao, Yun-Shan Lu, Tai-Fen Lee, Po-Ren Hsueh, Yu-Tsung Huang, Chun-Hsing Liao

**Affiliations:** 1 Department of Laboratory Medicine, National Taiwan University Hospital 38006 , Taipei, Taiwan; 2 Division of Infectious Diseases, Department of Internal Medicine, China Medical University, School of Medicine 665159 , Taichung, Taiwan; 3 Department of Laboratory Medicine, China Medical University, School of Medicine 665159 , Taichung, Taiwan; 4 School of Medicine, College of Medicine, National Yang Ming Chiao Tung University 210821 , Taipei, Taiwan; 5 Division of Infectious Diseases, Department of Internal Medicine, Far Eastern Memorial Hospital 46608 , New Taipei City, Taiwan; NorthShore University HealthSystem Department of Pathology and Laboratory Medicine, Evanston, Illinois, USA

**Keywords:** BluePoint MoldID, MALDI-TOF MS, filamentous fungi

## Abstract

BluePoint MoldID can identify 43 fungal species through nucleic acid array hybridization and matrix-assisted laser desorption ionization–time of flight mass spectrometry (MALDI-TOF MS) can identify 247 filamentous fungi through mass spectrometry. First, 43 standard isolates from the Bioresource Collection and Research Center, Taiwan, and the College of American Pathologists and 41 clinical *Aspergillus* species isolates confirmed by rDNA-ITS sequencing were analyzed using BluePoint MoldID and Bruker MALDI-TOF MS. BluePoint MoldID accurately identified 79% (34/43) of the standard isolates to the species level but failed to recognize nine isolates absent from its database; additionally, 87.8% (36/41) of the clinical isolates were identified at the species level, with 80.5% (33/41) accuracy. MALDI-TOF MS identified 86% (37/43) of the standard isolates, with 81.4% (35/43) accurately identified at the species level and two at the genus level, and identified all the clinical isolates, with 92.6% (38/41) accurately identified at the species level. Next, we analyzed 93 clinical *Aspergillus* species and compared the results by rDNA-ITS sequencing. BluePoint MoldID identified 87.1% (81/93) of the isolates at the species level, with 80.6% (75/93) accuracy. MALDI-TOF MS identified 97.8% (91/93) of the isolates, including some uncommon species, with 90.3% (84/93) accuracy at the species level. BluePoint MoldID and MALDI-TOF MS had turnaround times of 8 and 2 h, respectively, significantly reducing the time needed to identify filamentous fungi.

**IMPORTANCE:**

The BluePoint MoldID kit is an oligonucleotide array used for the identification of filamentous fungi, and it has not yet been mentioned in recent studies. We used a BluePoint MoldID kit to identify standard and clinical filamentous fungal isolates and compared its performance with that of Bruker MALDI-TOF MS. The former accurately identified 80.2% of the isolates (142/177), and the latter identified 92.6% of the isolates (164/177). The performance of the BluePoint MoldID kit was slightly inferior to that of Bruker MALDI-TOF MS because of the smaller database. However, the BluePoint MoldID kit can cover most clinically common opportunistic fungal infections; thus, it offers an alternative method for laboratories that lack MALDI-TOF MS equipment, as the device is less expensive.

## INTRODUCTION

Filamentous fungi comprise a diverse collection of pathogens that cause superficial and systemic fungal infections, and invasive fungal infections are a main cause of morbidity and mortality in hospitalized individuals. The worldwide incidence of fungal infections appears to be increasing (1). Infections caused by *Aspergillus* are associated with high mortality rates among hospitalized patients, especially those who are immunocompromised or suffer from serious underlying diseases (2). Treatments for fungal infections are based on initiating timely and effective antifungal therapy. However, the identification of filamentous fungi in a clinical laboratory still relies heavily on traditional phenotypic methods, which require several days of incubation and immense proficiency in fungal identification. The identification of fungi typically depends mainly on microscopic characteristics, which are intricate and time-consuming to analyze. Rapid and reliable identification of fungal pathogens is essential for providing suitable antifungal treatments to patients in a timely manner (2).

*Aspergillus* species are ubiquitous in the environment and are thus unavoidable (3). Invasive aspergillosis remains the dominant invasive fungal disease (IFD) among patients with hemato-oncological diseases and solid-organ transplant recipients and continues to increase yearly (4). Until the early 2000s, fewer than 10 species were considered clinically relevant. However, the ability to identify species has advanced owing to DNA sequencing analysis, which has revealed cryptic species that were previously unknown (5). Studies have shown that some rare/cryptic *Aspergillus* species isolates present antifungal susceptibility patterns that differ from those of classical pathogenic *Aspergillus* species (6). Therefore, the ability to identify *Aspergillus* species precisely is important for clinical diagnosis and antifungal treatment. Another method that has increased the ability to identify cryptic *Aspergillus* species is proteome fingerprint analysis by matrix-assisted laser desorption ionization–time of flight mass spectrometry (MALDI-TOF MS). MALDI-TOF MS shows a significant potential to bridge the existing gap in mycology diagnosis, as the method has altered practices in clinical bacteriology (7, 8). The widespread adoption of MALDI-TOF MS in clinical microbiology laboratories has enabled the swift and precise identification of various bacterial and yeast species (9, 10).

The BluePoint MoldID kit (Bio Concept Inc., Taichung, Taiwan) is a method for testing nucleic acids. Nucleic acid extraction is performed on filamentous fungi cultured in liquid medium, followed by polymerase chain reaction (PCR) to detect targets. After hybridization with a mold identification test device, The CHR113 color analysis instrument is used for photometric analysis, automatically determining if hybridization has occurred for array targets. The BluePoint analyzer is a photometric analysis instrument that uses a camera lens to capture the colored dots on the microbial identification test chip. The user can manually confirm the test results on the test chip on the basis of the image captured by the instrument for clinical identification.

This mold identification kit can identify 43 common filamentous fungi (Supplementary data 1) that cause respiratory and ocular infections, providing accurate species names for clinical physicians. In terms of testing time, fully mature fungi are not needed, and no subculturing is needed. Nucleic acid extraction can be performed without waiting for complete fungal growth, leading to a significant reduction in the identification precultivation period. Although the nucleic acid extraction and PCR steps are intricate, the entire identification process using the BluePoint mold identification kit can be completed in approximately 8 h, allowing prompt delivery of the necessary reports.

In this study, we explored two distinct new methods for fungal testing, namely, BluePoint MoldID and MALDI-TOF MS, with the goal of accelerating and accurately identifying clinical molds. A commercially available fungal database (MBT Filamentous Fungi Library 4.0) was developed for the Bruker Biotyper MALDI-TOF MS system for identifying clinical isolates of filamentous fungi grown in liquid medium using employing an extraction process. Currently, the database includes 247 common pathogenic filamentous fungi and has potential for future expansion (11, 12).

## MATERIALS AND METHODS

### Filamentous fungal isolates

**Fig 1:**
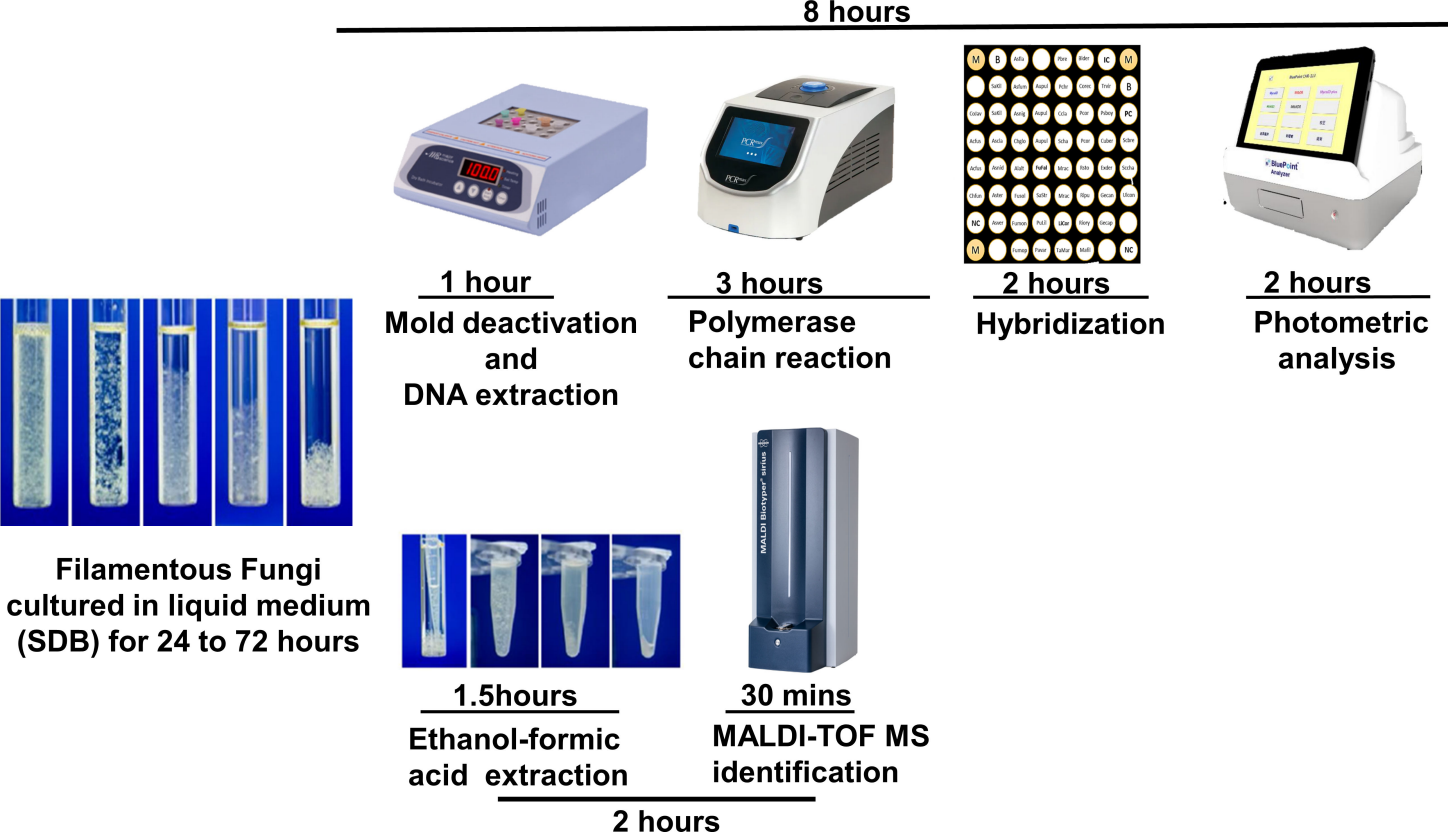
Procedures used for the BluePoint MoldID Kit and MALDI-TOF MS. The filamentous fungi were incubated in SDB medium and placed on a rotating platform at 25°C. The samples were then subjected to nucleic acid extraction and amplification via BluePoint MoldID and ethanol–formic acid extraction via MALDI-TOF identification.

We analyzed 84 isolates, comprising 43 standard isolates from the Bioresource Collection and Research Center (BCRC) and the College of American Pathologists, and 41 clinical *Aspergillus* species isolates confirmed by rDNA-ITS sequencing. The 43 standard isolates included *Aspergillus* species, *Blastomyces* species, *Chaetomium* species, *Fusarium* species, *Lichtheimia* species, *Malbranchea* species, *Microsporum* species, *Mucor* species, *Paecilomyces* species, *Penicillium* species, *Purpureocillium* species*, Rhizomucor* species, *Rhizopus* species, *Sarocladium* species, *Scedosporium* species, *Scopulariopsis* species, *Sporothrix schenckii* complex, *Stachybotrys* species, *Talaromyces* species, *Trichoderma* species, and *Trichophyton* species. The 41 clinical isolates included *Aspergillus flavus, Aspergillus fumigatus, Aspergillus unguis, Aspergillus niger, Aspergillus terreus, Aspergillus sydowii, Aspergillus caesiellus, Aspergillus hortai*, and *Aspergillus tabacinus*. In addition, we collected 93 *Aspergillus* species isolates from August 2023 to February 2024 that were cultured in our microbiology laboratory at NTUH. These samples were subjected to 24–72 h of incubation, followed by separate analyses using the BluePoint MoldID and Bruker MALDI-TOF MS (Fig. 1).

### Sample preparation

A filamentous fungal colony was added to Sabouraud dextrose broth (SDB) (Bio Star, #8201064) culture medium, which was placed on a rotating platform and incubated at 25°C until sufficient growth was observed. BSL-2 and ABSL-2 practices, containment equipment, and facilities are recommended when working with clinical samples, animal tissues, yeast-form cultures, and infected animals. BSL-3 practices, containment equipment, and facilities are required for handling sporulating mold-form cultures identified as dimorphic fungal pathogens and for soil or other environmental samples that are known or likely to contain infectious conidia.

### Identification with the BluePoint MoldID kit

The BluePoint MoldID kit (Bio Concept Inc., Taichung, Taiwan #867405) is a qualitative test involving molecular biological technology. It was approved by the Taiwan Food and Drug Administration MOHW-MD-(I)-No.005274 and is available for *in vitro* diagnosis. For nucleic acid extraction, we added filamentous fungal samples to DNA extraction tubes and vortexed them for 5 s. For each tube, the mixture was subsequently centrifuged at 13,500 rpm for 5 min, after which the supernatant was discarded. After 50 µL of lysis buffer was added and vortexed vigorously for 10 min to disrupt the mold cells, the cells were heated to 95°C for 5 min, and 100 µL of neutralizer buffer was added. The mixture was subsequently centrifuged at 13,500 rpm for 5 min, after which the supernatant was collected as the DNA sample. The DNA sample should be stored at −20℃ no longer than 3 days. After nucleic acid extraction, PCR was conducted with specific primers following the manufacturer’s instructions. The PCR products which labeled with biotin were stored on ice for subsequent reverse dot-blot hybridization immediately or stored at −20℃ no longer than 7 days. The BluePoint MoldID device is a chip that contains different hybridization dots. The hybridization reactions were performed following the manufacturer’s instructions. Then, we added the streptavidin-conjugated latex reagent, and the dots on the chip developed color, which could be interpreted visually or by a CHR113 color reader. The interpretation of the results must be completed within 24 h after the photometric reaction.

### MALDI-TOF MS identification

For each culture, the tube was removed from the rotating platform and left upright for 10 min, after which 1.5 mL of the precipitate was transferred to a microcentrifuge tube. The mixture was subsequently centrifuged at 13,000 rpm for 2 min, after which the supernatant was removed. One milliliter of deionized water was added, and the mixture was vortexed for 1 min and centrifuged at 13,000 rpm for 2 min. The supernatant was removed, deionized water was added, and the sample was centrifuged to remove the supernatant. The precipitate was dispersed by adding 300 µL of distilled water, 900 µL of absolute ethanol was added, and the mixture was vortexed thoroughly. The sample was centrifuged at 13,000 rpm for 2 min to remove the supernatant and subsequently centrifuged again with a micropipette to remove as much of the remaining supernatant as possible. The samples were completely air-dried using a vacuum concentrator or air-dried at 37°C for approximately 5–10 min. Approximately 10–20 or 100 µL of 70% formic acid (Sigma‒Aldrich) was added to the precipitate, and an equal volume of acetonitrile (J.T. Baker) was added to the 70% FA solution. Zirconia/silica lysis beads (BioSpec, #11079105z) were added, and the mixture was vortexed vigorously for 10 min to break the cell walls and then centrifuged at 13,000 rpm for 2 min.

### MALDI-TOF MS spectral identification

One microliter of the supernatant was placed on a MALDI sample plate (Bruker Daltonics GmbH, Bremen, Germany) and air-dried at room temperature. Then, 1 µL of α-cyano-hydroxycinnamic acid (HCCA) matrix solution (Bruker Daltonics GmbH) was placed on the MALDI sample plate and air-dried before MALDI-TOF analysis. The MALDI-TOF mass spectra were automatically compared with those of the Bruker MBT Filamentous Fungi Library version 4.0, which covers 247 species/species groups.

### MALDI-TOF MS spectral analysis

In accordance with the Bruker recommendations, the level of similarity between an unknown tested sample and a reference sample is indicated by a log(score), which is referred to herein as a “score.” A score of >2.3 indicates “highly probable species identification”; a score of >2 and <2.299 indicates “secure genus identification, probable species identification”; a score of >1.7 and <1.999 indicates “probable genus identification”; and a score of <1.7 indicates “unreliable identification.” However, these score thresholds have been designed for bacterial identification and are not necessarily appropriate for fungal identification. Several studies have analyzed the impact of lowering the cutoff level on the number of correct identifications (13). Lowering the cutoff levels to ≥1.7 for species-level identifications and ≥1.5 for genus-level identifications resulted in an increase in accurate species-level identifications (14, 15), and no increase in the number of misidentifications was reported (16). These results show that reliable species identification via MALDI-TOF MS can also be achieved by using a log score of 1.7. Therefore, we set the criterion for correct species identification at ≥1.7 in this study.

### Reference identification of isolates

In addition to identification using phenotypic and growth characteristics, sequence analysis was performed for the molecular identification of most isolates. The internal transcribed spacer (17) region of the 18S to 26S rDNA gene was amplified, and sequence analysis was used for species-level identification (18, 19). The sequences of the forward primer used was (5′-GCATCGATGAAGAACGCAGC-3′) and reverse primer used was (5′-TCCTCCGCTTATTGATATGC-3′). The calmodulin gene was amplified using forward primer cmd5 (5′-CCGAGTACAAGGAGGCCTTC-3′) and reverse primer cmd6 (5′-CCGATAGAGGTCATAACGTGG-3′). The PCR conditions were as follows: initial denaturation at 95°C for 5 min, followed by 35 × 95°C for 30 s, 58°C for 30 s, 72°C for 1 min, and 72°C for 10 min for the final extension reaction.

## RESULTS

### Performance of BluePoint MoldID and MALDI-TOF MS in the identification of standard strains and sequencing of *Aspergillus* isolates

For 43 standard strains, the BluePoint MoldID successfully identified 34 strains (79%) at the species level. The remaining nine isolates that were not recognized from the database, including *Aspergillus ustus*, *Microsporum gypseum*, *Mucor circinelloides, Sporothrix schenckii* complex, and *Trichophyton tonsurans*. Although some tested isolates were not listed in the database of BluePoint MoldID, in practice, we do not actually know what the infectious strain is supposed to be when the BluePoint MoldID is used on clinical specimens; therefore, we intentionally tested random samples to observe the potential clinical performance.

MALDI-TOF MS successfully identified 86% (37/43) of the standard isolates, with 35 strains (81.4%) accurately identified to the species level, whereas the remaining two isolates (*Fusarium oxysporum* and *Pseudallescheria boydii* complex) were identified to the genus level. MALDI-TOF failed to identify the following six species: *Fusarium falciforme*, *Blastomyces dermatitidis*, *Malbranchea filamentosa*, *Purpureocillium lilacinus*, *Talaromyces marneffei*, and *Scopulariopsis chartarum* (Table 1). For the 41 sequenced clinical isolates of *Aspergillus* species, BluePoint MoldID identified 87.8% (36/41) of the isolates, with 80.5% (33/41) accurately identified to the species level. The five isolates that were not identified were *Aspergillus flavus* (1)*, Aspergillus unguis* (1)*, Aspergillus niger* (2), and *Aspergillus tabacinus* (1). The three isolates for which MoldID was identified at the genus level but not at the species level were *Aspergillus caesiellus*, *Aspergillus hortai* and *Aspergillus terreus*. MALDI-TOF MS identified all 41 strains, with 92.6% (38/41) accurately identified to the species level. The three isolates for which MALDI-TOF MS failed to identify at the species level were *Aspergillus caesiellus*, *Aspergillus hortai*, and *Aspergillus tabacinus*, which were not listed in the MALDI-TOF database (Table 2). Among the 84 filamentous fungal isolates, 79.7% (67/84) were identified by BluePoint MoldID, and 86.9% (73/84) were identified by MALDI-TOF MS to the species level. The turnaround times for the MoldID kit and MALDI-TOF MS were 8 and 2 h, respectively. Both methods significantly reduced the time needed to identify filamentous fungi. However, as the BluePoint MoldID is limited to 43 strains, MALDI-TOF MS exhibited a superior identification performance because of its broader database and expansion potential.

**TABLE 1:** Identification results obtained for the clinical isolates using BluePoint MoldID kit and MALDI-TOF MS^*a*^

Standard strains (*n* = 43)	Methods	Level of identification		
		Species	Genus	No ID
*Aspergillus* spp. (*n* = 15)	BluePoint Mold ID	13	0	2
	MALDI-TOF MS	15	0	0
*Absidia* spp. (*n* = 1)	BluePoint Mold ID	1	0	0
	MALDI-TOF MS	1	0	0
*Acremonium* spp. (*n* = 2)	BluePoint Mold ID	2	0	0
	MALDI-TOF MS	1	0	1
*Blastomyces* spp. (*n* = 1)	BluePoint Mold ID	1	0	0
	MALDI-TOF MS	0	0	1
*Chaetomium* spp. (*n* = 1)	BluePoint Mold ID	1	0	0
	MALDI-TOF MS	1	0	0
*Fusarium* spp. (*n* = 1)	BluePoint Mold ID	1	0	0
	MALDI-TOF MS	1	0	0
*Malbranchea* spp. (*n* = 1)	BluePoint Mold ID	1	0	0
	MALDI-TOF MS	0	0	1
*Microsporum* spp. (*n* = 4)	BluePoint Mold ID	0	0	4
	MALDI-TOF MS	4	0	0
*Mucor* spp. (*n* = 2)	BluePoint Mold ID	1	0	1
	MALDI-TOF MS	2	0	0
*Paecilomyces* spp. (*n* = 2)	BluePoint Mold ID	2	0	0
	MALDI-TOF MS	1	0	1
*Penicillium* spp. (*n* = 4)	BluePoint Mold ID	4	0	0
	MALDI-TOF MS	3	0	1
*Scedosporium* spp. (*n* = 1)	BluePoint Mold ID	1	0	0
	MALDI-TOF MS	0	1	0
*Rhizomucor* spp. (*n* = 1)	BluePoint Mold ID	1	0	0
	MALDI-TOF MS	1	0	0
*Rhizopus* spp. (*n* = 1)	BluePoint Mold ID	1	0	0
	MALDI-TOF MS	1	0	0
*Scopulariopsis* spp. (*n* = 2)	BluePoint Mold ID	2	0	0
	MALDI-TOF MS	1	0	1
*Sporothrix schenckii* complex (*n* = 1)	BluePoint Mold ID	0	0	1
	MALDI-TOF MS	1	0	0
*Stachybotrys* spp. (*n* = 1)	BluePoint Mold ID	1	0	0
	MALDI-TOF MS	1	0	0
*Trichoderma* spp. (*n* = 1)	BluePoint Mold ID	1	0	0
	MALDI-TOF MS	0	1	0
*Trichophyton* spp. (*n* = 1)	BluePoint Mold ID	0	0	1
	MALDI-TOF MS	1	0	0
Total (*n* = 43)	BluePoint Mold ID	34	0	9
	MALDI-TOF MS	35	2	6

^*a*^ Forty-three isolates of standard strains were identified by BluePoint MoldID and MALDI-TOF. The BluePoint MoldID identified 34 isolates to the species level, 9 of which were not identified. MALDI-TOF was used to identify 35 isolates at the species level and 2 isolates at the genus level, and 6 of these isolates could not be identified.

**TABLE 2:** Results for the identification of the clinical isolates using MALDI-TOF MS and the BluePoint MoldID kit^*a*^

*Aspergillus* species (*n* = 41)	Methods	Level of identification		
		Species	Genus	No ID
*A. flavus* (*n* = 7)	BluePoint Mold ID	6	0	1
	MALDI-TOF MS	7	0	0
*A. fumigatus* (*n* = 5)	BluePoint Mold ID	5	0	0
	MALDI-TOF MS	5	0	0
*A. unguis* (*n* = 1)	BluePoint Mold ID	0	0	1
	MALDI-TOF MS	1	0	0
*A. niger* (*n* = 11)	BluePoint Mold ID	9	0	2
	MALDI-TOF MS	11	0	0
*A. terreus* (*n* = 10)	BluePoint Mold ID	9	1	0
	MALDI-TOF MS	10	0	0
*A. sydowii* (*n* = 4)	BluePoint Mold ID	2	2	0
	MALDI-TOF MS	4	0	0
*A. caesiellus* (*n* = 1)	BluePoint Mold ID	0	1	0
	MALDI-TOF MS	0	1	0
*A. hortai* (*n* = 1)	BluePoint Mold ID	0	1	0
	MALDI-TOF MS	0	1	0
*A. tabacinus* (*n* = 1)	BluePoint Mold ID	0	0	1
	MALDI-TOF MS	0	1	0
Total (*n* = 41)	BluePoint Mold ID	31	5	5
	MALDI-TOF MS	38	3	0

^*a*^ Forty-one isolates of *Aspergillus* species were identified by BluePoint MoldID and MALDI-TOF. The BluePoint MoldID identified 31 isolates to the species level and 5 isolates to the genus level, with 5 isolates that were not identified. MALDI-TOF identified 38 isolates at the species level and 3 isolates at the genus level.

### Comparison of BluePoint MoldID and MALDI-TOF MS for the identification of clinically unknown *Aspergillus* species

**Fig 2:**
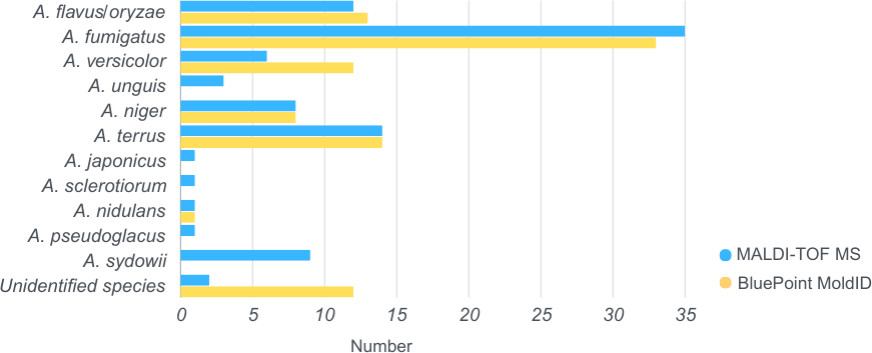
The 93 *Aspergillus* isolates identified by The BluePoint MoldID kit and MALDI-TOF MS. The yellow bar shows the results obtained for the BluePoint MoldID, and the blue bar shows the results obtained for MALDI-TOF.

We additionally collected 93 clinical isolates of *Aspergillus* species from the microbiology laboratory at the NTUH to confirm their ability to identify *Aspergillus* species. These samples were analyzed by the two different platforms simultaneously, and the results were compared. The samples were subjected to rDNA-ITS sequencing if there were any differences between the BluePoint MoldID and MALDI-TOF MS results. In these tests, BluePoint MoldID identified *Aspergillus flavus/oryzae* (13), *Aspergillus fumigatus* (33), *Aspergillus versicolor* (12), *Aspergillus niger* (8), *Aspergillus terreus* (14), *Aspergillus nidulans* (1), and 12 unidentified species. Similarly, MALDI-TOF MS identified *Aspergillus flavus/oryzae* (12), *Aspergillus fumigatus* (35), *Aspergillus versicolor* (6), *Aspergillus sydowii* (9), *Aspergillus unguis* (3), *Aspergillus niger* (8), *Aspergillus terreus* (14), *Aspergillus japonicus* (1), *Aspergillus sclerotiorum* (1), *Aspergillus nidulans* (1), *Aspergillus pseudoglaucus* (1), and two unidentified species (Fig. 2). There were 74 identical results in MALDI-TOF MS and BluePoint MoldID, whereas there were 19 discrepant results among the 93 samples, and these 19 isolates were subsequently subjected to rDNA-ITS sequencing. These discrepant results are listed in Table 3 and were compared with the sequencing results. The sequencing results were as follows: *A. unguis* (2), *A. fumigatus* (4), *A. aculeatus* (1), *A. versicolor* (2), *A. sydowii* (4), *A. caesiellus* (2), *A. sclerotiorum* (1), *A. ochraceopetaliformis* (1), and *A. flocculosus* (2). The nine MALDI‒TOF MS results correspond to the sequence data, whereas only the one BluePoint MoldID result corresponds to the sequence data. Among the 18 isolates that were not identified by BluePoint MoldID, 13 were not listed in the database, including *A. unguis, A. aculeatus, A. sydowii, A. caesiellus, A. sclerotiorum, A. ochraceopetaliformis,* and *A. flocculosus.* Among the other five isolates, BluePoint MoldID failed to identify three *A. fumigatus* isolates and one *A. versicolor* isolate and misidentified an *A. fumigatus* isolate as *A. flavus/oryzae.* Among the 10 isolates not identified by MALDI-TOF MS, six were not listed in the database, namely, *A. aculeatus, A. caesiellus, A. ochraceopetaliformis,* and *A. flocculosus.* MALDI-TOF MS misidentified two *A. fumigatus* isolates as *A. sydowii* and *A. unguis* and misidentified two *A. versicolor* isolates as *A. sydowii.* For the 93 *Aspergillus* species isolates, BluePoint MoldID accurately identified 80.6% (75/93) of the isolates, and MALDI-TOF MS accurately identified 90.3% (84/75) of the isolates at the species level. The performance of the two different methods for identifying unknown *Aspergillus* isolates was consistent with their performance in identifying standard and sequenced isolates.

**TABLE 3:** rDNA-ITS sequencing results of the 19 discrepant MALDI-TOF MS and BluePoint MoldID results for 93 clinical isolates

	rDNA-ITS	MALDI-TOF MS	BluePoint Mold ID
1	*Aspergillus unguis*	*Aspergillus unguis*	Unidentified species^*a*^
2	*Aspergillus fumigatus*	*Aspergillus fumigatus*	*Aspergillus flavus/oryzae^a^*
3	*Aspergillus aculeatus*	*Aspergillus japonicus^a^*	Unidentified species^*a*^
4	*Aspergillus versicolor*	*Aspergillus sydowii^a^*	Unidentified species^*a*^
5	*Aspergillus sydowii*	*Aspergillus sydowii*	Unidentified species^*a*^
6	*Aspergillus caesiellus*	*Aspergillus sydowii^a^*	*Aspergillus versicolor^a^*
7	*Aspergillus sclerotiorum*	*Aspergillus sclerotiorum*	Unidentified species^*a*^
8	*Aspergillus fumigatus*	*Aspergillus unguis^a^*	Unidentified species^*a*^
9	*Aspergillus fumigatus*	*Aspergillus sydowii^a^*	Unidentified species^*a*^
10	*Aspergillus caesiellus*	*Aspergillus sydowii^a^*	*Aspergillus versicolor^a^*
11	*Aspergillus fumigatus*	*Aspergillus fumigatus*	Unidentified species^*a*^
12	*Aspergillus sydowii*	*Aspergillus sydowii*	*Aspergillus versicolor^a^*
13	*Aspergillus ochraceopetaliformis*	*Aspergillus pseudoglaucus^a^*	Unidentified species^*a*^
14	*Aspergillus unguis*	*Aspergillus unguis*	Unidentified species^*a*^
15	*Aspergillus sydowii*	*Aspergillus sydowii*	*Aspergillus versicolor^a^*
16	*Aspergillus versicolor*	*Aspergillus sydowii^a^*	*Aspergillus versicolor*
17	*Aspergillus flocculosus*	Unidentified species^*a*^	Unidentified species^*a*^
18	*Aspergillus flocculosus*	Unidentified species^*a*^	Unidentified species^*a*^
19	*Aspergillus sydowii*	*Aspergillus sydowii*	*Aspergillus versicolor^a^*

^*a*^ Inconsistent MALDI-TOF MS and BluePoint MoldID results with rDNA-ITS sequencing.

## DISCUSSION

In the first part of this study, we investigated two different methods for the identification of filamentous fungi to shorten the turnaround time. During the identification of standard isolates, both BluePoint MoldID and MALDI-TOF MS identified the species listed in their databases but failed to identify the species absent from their database. During the identification of the sequenced clinical *Aspergillus* isolates, BluePoint MoldID failed to identify some common pathogens, such as *Aspergillus niger* and *Aspergillus terreus*. Since the BluePoint MoldID method involves PCR and hybridization reactions, the presence of inhibitory substances in the sample may affect the test results. Additionally, when the target gene sequence of the samples undergoes mutations or variations, it may not hybridize with the oligonucleotide gene probe on the test chip, leading to a false-negative result. MALDI-TOF MS failed to identify *Aspergillus caesiellus*, *Aspergillus hortai*, and *Aspergillus tabacinus*, which are not commonly isolated from clinical specimens and were not listed in its database. According to a study on the taxonomy of *Aspergillus* species, the taxonomic rearrangements of the series Versicolores (20), *Aspergillus tabacinus* and *Aspergillus versicolor* were synonymous; therefore, MALDI-TOF MS identified *Aspergillus tabacinus* as *Aspergillus versicolor*. Furthermore, *Aspergillus hortai* and *Aspergillus terreus* share a close biological relationship based on analyses of β-tubulin and calmodulin sequence data in previous studies in Section Terrei of *Aspergillus* (21). This might explain why MALDI-TOF MS identified *Aspergillus hortai* as *Aspergillus terreus*.

MALDI-TOF MS has already changed fungal identification strategies because of its fast identification process. In contrast to MALDI-TOF MS, which analyzes protein spectra, the BluePoint MoldID kit uses a chip hybridization assay to identify nucleic acids. We evaluated two different methods for identifying filamentous fungi in a clinical laboratory. Compared with the BluePoint MoldID kit, MALDI-TOF MS performed better for the identification of *Aspergillus* isolates; however, the methods performed equally well for standard isolates. The BluePoint MoldID system misidentified an *Aspergillus tabacinus* isolate as *Cladosporium cladosporioides*, while MALDI-TOF MS identified this isolate to the genus level. Moreover, one *Aspergillus flavus*, one *Aspergillus unguis*, and two *Aspergillus niger* isolates were not detected by the BluePoint MoldID kit, whereas the MALDI-TOF MS system correctly identified these isolates to the species level. The BluePoint MoldID kit may have misidentified this isolate because the target gene sequences of the tested fungi were similar, leading to cross-hybridization reactions of the oligonucleotide gene probes and resulting in identification errors. The limitation in identifying filamentous fungi through the BluePoint MoldID system involves insufficient coverage of species in reference databases.

In the second part of this study, we aimed to identify *Aspergillus* species, which are the most common ubiquitous opportunistic pathogens (22). We collected 93 clinical isolates that were identified as *Aspergillus* species through microscopic screening and simultaneously analyzed them by MALDI-TOF MS and BluePoint MoldID. BluePoint MoldID revealed 12 unidentified isolates, while MALDI-TOF MS revealed two unidentified isolates. The two different methods generated contrasting results for seven isolates. To analyze the accuracy of the two different methods, rDNA-ITS sequencing was conducted on these 19 isolates. The sequencing results revealed several uncommonly observed *Aspergillus* species, such as *A. aculeatus, A. caesiellus, A. ochraceopetaliformis,* and *A. flocculosus*, which are not listed in the MALDI-TOF MS or BluePoint MoldID databases. *A. aculeatus* and *A. japonicus* are uniseriate groups of black aspergilli and are defined in the *Aspergillus* section *Nigri* (23). The DNA sequences of the internal transcribed spacers (ITS1 and ITS2) and the 5.8S rRNA gene could not be used to distinguish between *A. japonicus* and *A. aculeatus*. These two taxa are more closely related to each other than to other species of black aspergilli (24), which may lead to misidentification by MALDI-TOF. The microscopic structure and molecular phylogeny of *A. caesiellus* are similar to those of *A. sydowii* and *A. versicolor* because of the D1–D2 domain of the 28S rDNA gene (25). This might explain why MALDI-TOF MS misidentified *A. caesiellus* and *A. versicolor* as *A. sydowii* and misidentified *A. versicolor* as *A. sydowii.* The BluePoint MoldID database contains *A. versicolor* but not *A. sydowii* or *A. caesiellus*; therefore, the method failed to identify *A. sydowii* and *A. caesiellus* or identify them as *A. versicolor*. According to recent investigations on the taxonomy of *Aspergillus* species and phylogenetic molecular analyses (26), *A. ochraceopetaliformis* was found to be conspecific with *A. flocculosus* in section *Circumdati*; these results were based on phylograms derived from four combined loci, beta tubulin (BT2), calmodulin (CF), ITS and lsu rDNA (ID), and RNA polymerase II (RPB2) (27, 28). *A. ochraceopetaliformis* and *A. pseudoglaucus* have been linked to cutaneous aspergillosis (27, 29). These rare *Aspergillus* species, which cannot be identified by MALDI-TOF MS or BluePoint MoldID, can be distinguished only by sequencing specific loci. *A. unguis,* which was identified by MALDI-TOF MS but is not listed in the BluePoint database, was reported to be a cryptic species that causes invasive fungal disease (30) and retained low triazole MIC values, which is different from other cryptic species. This finding demonstrated the importance of correctly identifying rare cryptic species. Moreover, BluePoint MoldID failed to identified four ITS2-confirmed *A. fumigatus*, which is of significant medical importance. To reveal if these isolates are cryptic species, the 19 discrepancy isolates were subjected to calmodulin sequencing. (Supplementary data 3). However, the results of calmodulin sequencing in the four *A. fumigatus* isolates were identical to ITS sequencing. Therefore, the BluePoint MoldID failed to identify these *A. fumigatus* isolates may owing to the variation or mutations in the probe hybridization sequence.

Currently, DNA sequencing is the gold standard for identifying filamentous fungi at the species level. Despite their accuracy, DNA sequencing methods are time consuming, require specialized equipment or expertise and are not typically accessible in clinical laboratories. Using MALDI-TOF MS to identify filamentous fungi, especially those that are rare, cryptic, or morphologically indistinguishable, may be a valid alternative to DNA sequence-based identification (31). The latter method is time-consuming because β-tubulin (benA or tub-2) and calmodulin (12) must be analyzed as secondary genetic markers, as well as the internal transcribed spacer (17) region.

The database of the BluePoint MoldID kit includes 43 species of respiratory pathogens, mostly *Aspergillus* species, *Fusarium* species and *Penicillium* species, but does not include common dermatophytes, such as *Microsporum* species and *Trichophyton* species. These fungi may cause severe and disseminated infections in immunocompromised hosts (32). However, a MALDI-TOF MS system equipped with a commercial database can correctly identify these dermatophytes. There are no reports on the performance of the BluePoint MoldID kit in the identification of filamentous molds. In our study, the BluePoint MoldID kit showed over 80% accuracy in identifying both standard isolates and clinical *Aspergillus* isolates, while MALDI-TOF MS accurately identified more than 85% of the standard isolates and more than 90% of the clinical *Aspergillus* isolates. However, during the identification of standard isolates, the BluePoint MoldID kit accurately identified the following six species that MALDI-TOF MS failed to identify: *Fusarium falciforme*, *Blastomyces dermatitidis*, *Malbranchea filamentosa*, *Purpureocillium lilacinus*, *Talaromyces marneffei*, and *Scopulariopsis chartarum*. Another PCR-based nucleic acid amplification test, DNA barcoding, has been applied for identifying filamentous fungi. Ollinger et al*.* presented a combination of eight primer pairs to identify molds within a single PCR run. The method successfully identified *Penicillium chrysogenum*, *Penicillium citrinum*, *Cladosporium sphaerospermum*, *Paecilomyces formosus*, *Rhizopus oryzae*, and *Aspergillus niger* from samples collected at a local bakery (17). However, the effectiveness of this method relies heavily on the DNA extraction methods employed and, crucially, on the primer pairs selected to target specific DNA sequences. A major problem for fungal identification is the lack of universal primers (33). Liao et al*.* developed a microsphere-based suspension array (MSA) by designing 21 oligonucleotide probes based on the internal transcribed spacer 2 (ITS2) region for identifying filamentous fungi at the species level (34). The MSA assay can be applied for multiplexed identification of 23 species of clinically relevant filamentous molds and dimorphic fungi. Moreover, Hung et al*.* developed an oligonucleotide array that could accurately identify 21 important airborne fungi that may cause adverse health problems (35). In the current method, the key feature is the standardized protocol, which includes DNA extraction, ITS amplification, and membrane hybridization. Moreover, the oligonucleotide array identified multiple fungal species simultaneously when two or three species were mixed and cultured on agar plates. The BluePoint MoldID kit was derived from oligonucleotide array studies (34, 35), and its database was expanded to 43 species. In testing 155 target reference strains, the sensitivity of the BluePoint MoldID kit is 94%, and the specificity is 91%. The BluePoint MoldID database was established using ITS1 and ITS4, and the most recent review was conducted in 2022, and it was also updated in the manual simultaneously. Unlike a traditional glass chip, the BluePoint MoldID kit uses a membrane array, which is simpler, more time-efficient, and significantly less expensive. The only instrumentation needed for this method is a shaker and an incubator for hybridization.

The major limitation of MALDI-TOF MS for mold identification is the limited scope of commercially available databases. This constraint has hindered the widespread adoption of MALDI-TOF MS in clinical laboratories for identifying filamentous fungi. In 2020, Hankins et al*.* compared the following mass spectrometry systems used in hospital laboratories to identify molds from patient samples: Vitek MS (v. 3.0) using MylaLab software (v. 4.8.2-0), Bruker Biotyper systems using MBT Compass software (v. 4.0.19) and the MBT Filamentous Fungi Database (v. 4.0) (36). The authors reported that for the species included in the manufacturers’ databases, Vitek MS correctly identified 85% of the isolates and misidentified 8%; the Bruker Biotyper identified 64% of the isolates accurately, with no misidentification. For isolates not in the databases, the Bruker Biotyper had no misidentifications, while the Vitek MS misidentified 36% of the isolates. Overall, compared with Vitek MS, the Bruker Biotyper achieved a greater percentage of accurate species-level identifications and had fewer misidentifications. Masih et al*.* achieved successful MALDI-TOF MS identification (score value of ≥2.0) for 97.7% of 45 clinically significant *Aspergillus* isolates using the Bruker Biotyper database (OC version 3.1; updated January 2016) supplemented with an in-house fungal database to improve the identification of *Aspergillus* species (37). These isolates belonged to 23 rare *Aspergillus* species, among which eight were included in the Bruker Biotyper database. Hamal et al*.* identified 193 isolates of filamentous fungi by Bruker MALDI-TOF MS directly from the culture after the samples were cultured in liquid medium with extraction. The agreement rates between identification using morphology and the MALDI-TOF MS direct and extraction methods were 58.55% and 84.97% at the genus level and 22.24% and 46.11% at the species level, respectively. Compared with sequencing, the MALDI-TOF MS extraction method showed an 87.5% agreement rate, while the direct method had only a 43.75% agreement rate (38). According to the studies above, MALDI-TOF MS better identified *Aspergillus* species than other filamentous fungi did. We observed a similar phenomenon in our study. Additionally, in 2011, Theel et al. used MALDI-TOF MS and a commercial library, and only 20.5% of dermatophyte isolates were correctly identified. However, when an additional 20 in-house dermatophyte spectra were added, the identification rate increased to 60% (39). As evidenced by previous studies (36–39), high identification rates with MALDI-TOF MS are achieved only when in-house reference spectrum databases are utilized. In this study, we focused on identifying *Aspergillus* species with the newest version of the commercial MBT Filamentous Fungi Database (v. 4.0), which contains 247 strains. Without an in-house database, MALDI-TOF MS identified 90.3% (84/93) of the *Aspergillus* species accurately at the species level. However, it is necessary to expand the MALDI-TOF MS database to address more complicated infections by filamentous fungi.

Owing to the high cost of mass spectrometer devices and commercial databases, MALDI-TOF MS systems are not ubiquitous in small-scale medical laboratories. Moreover, the method is time consuming and requires specialized equipment or expertise to expand the in-house spectrum database. BluePoint MoldID offers an alternative method for laboratories that lack MALDI-TOF MS equipment, as the device is less expensive and easier to use. Although the turnaround time of the BluePoint MoldID kit is longer than that of MALDI-TOF MS, technicians can more easily follow the directions of the BluePoint MoldID kit without high levels of expertise. The 43 filamentous fungal species in the BluePoint MoldID database can cover the identification of common respiratory and ocular infections (40, 41).

However, some limitations should be noted. First, the BluePoint MoldID involves PCR and hybridization reactions, the present of mutations or variations of target gene sequence may lead to a false-negative result. Second, at the time of BluePoint MoldID design, the importance of cryptic species was poorly understood, and its database was established using ITS genes, so the resolution is insufficient to identify some cryptic *Aspergillus* species. Finally, because of the small sample size and the fact that the specimens were from a single region, it was difficult to demonstrate the performance of BluePoint MoldID in other areas.

Considering the increasing prevalence of invasive fungal infections (42), a rapid method for identifying filamentous fungi is needed. Compared with BluePoint MoldID, MALDI-TOF MS can identify more *Aspergillus* species, including common and some cryptic species. The use of an in-house spectrum database can increase the number of species that can be identified by MALDI-TOF MS. However, the BluePoint MoldID kit, a commercial oligonucleotide array, could offer an alternative method in laboratories that lack MALDI-TOF abilities owing to its lower cost and minimal instrumentation, as well as its satisfactory identification rates and turnaround time.

## Data Availability

The data that support the findings of this study are available from corresponding author Y.-T. H. upon reasonable request.

## SUPPLEMENTAL MATERIAL

Supplemental material jcm.01048-24-s0001.docx Data S1 to S3.

ASM does not own the copyrights to Supplemental Material that may be linked to, or accessed through, an article. The authors have granted ASM a non-exclusive, world-wide license to publish the Supplemental Material files. Please contact the corresponding author directly for reuse.
