# Supplementary material for: Comparison of the BluePoint MoldID oligonucleotide array and Bruker Biotyper MALDI-TOF MS for the identification of filamentous fungi
Source: J Clin Microbiol. 2024 Dec 5;63(1):e01048-24. doi: 10.1128/jcm.01048-24 (PMC11784249; doi:10.1128/jcm.01048-24)
Supplement: Supplemental material — Data S1 to S3. [file jcm.01048-24-s0001.docx]

**Supplementary data 1**

The 43 species list of targets for the BluePoint^TM^ MoldID system.

| 1 | *Acrophialophora fusispora* |
| --- | --- |
| 2 | *Alternaria alternate* |
| 3 | *Aspergillus clavatus* |
| 4 | *Aspergillus flavus/* oryzae |
| 5 | *Aspergillus fumigatus* |
| 6 | *Aspergillus nidulans* |
| 7 | *Aspergillus niger* |
| 8 | *Aspergillus terreus* |
| 9 | *Aspergillus versicolor* |
| 10 | *Aureobasidium pullulans* |
| 11 | *Blastomyces dermatitidis* |
| 12 | *Chaetomium globosum* |
| 13 | *Chaetomium funicola* |
| 14 | *Cladosporium cladosporioides* |
| 15 | *Cordyceps javanica* |
| 16 | *Cunninghamella bertholletiae* |
| 17 | *Exophiala dermatitidis* |
| 18 | *Fusarium falciforme* |
| 19 | *Fusarium moniliforme* |
| 20 | *Fusarium oxysporum* |
| 21 | *Fusarium solani* |
| 22 | *Geotrichum candidum* |
| 23 | *Geotrichum capitatum* |
| 24 | *Lichtheimia corymbifera* |
| 25 | *Malbranchea filamentosa* |
| 26 | *Mucor racemosus* |
| 27 | *Paecilomyces variotii* |
| 28 | *Penicillium brevicompactum* |
| 29 | *Penicillium chrysogenum* |
| 30 | *Penicillium corylophilum* |
| 31 | *Purpureocillium lilacinum* |
| 32 | *Pseudallescheria boydii* |
| 33 | *Rhizomucor pusillus* |
| 34 | *Rhizopus oryzae* |
| 35 | *Rhizopus stolonifer* |
| 36 | *Sarocladium kiliense* |
| 37 | *Sarocladium strictum* |
| 38 | *Scopulariopsis brevicaulis* |
| 39 | *Scopulariopsis chartarum* |
| 40 | *Stachybotrys chartarum* |
| 41 | *Talaromyces marneffei* |
| 42 | *Trichoderma viride* |
| 43 | *Ulocladium consortiale* |

Because some strains have similar ITS sequences, if the following strains are detected, further distinction and identification should be performed using additional methods.

1. *Aspergillus versicolor* (*A. versicolor* and *A. sydowii* have similar ITS sequences)
2. *Cladosporium cladosporioides* (*C. cladosporioides*, *C. tenuissimum*, *C. colocasiae* and *C.oxysporum* have similar ITS sequences)
3. *Chaetomium fumicola* (*C. globosum, C. cochlioides* and *C. fumicola* have similar ITS sequences)
4. *Fusarium moniliforme* (*F. moniliforme, F. oxysporum* and *F. pallidoroseum* have similar ITS sequences)
5. *Paecilomyces variotii* (*P. variotii, P. formosus* and *P. alba* have similar ITS sequences)
6. *Penicillium chrysogenum* (*P. chrysogenum* and *P. commune* have similar ITS sequences)
7. *Talaromyces marneffei* (*T. marneffei* and *T. emersonii* have similar ITS sequences)
8. *Malbranchea filamentosa* (*M. filamentosa* and *M. setosa* have similar ITS sequences)

**Supplementary data 2**

The list reconciling matches and nonmatches between BluePoint MoldID and Bruker Biotyper MALDI-TOF MS. The underline name indicates the non-match names in the Bruker MALDI-TOF database.

The “－” symbol indicates that this species does not appear in the Bruker MALDI-TOF database.

| BluePoint MoldID database | Bruker MALDI-TOF datadase |
| --- | --- |
| *Acrophialophora fusispora* | － |
| *Alternaria alternate* | *Alternaria alternata* |
| *Aspergillus clavatus* | *Aspergillus clavatus* |
| *Aspergillus flavus* | *Aspergillus flavus* |
| *Aspergillus fumigatus* | *Aspergillus fumigatus* |
| *Aspergillus nidulans* | *Aspergillus nidulans* |
| *Aspergillus niger* | *Aspergillus niger* |
| *Aspergillus terreus* | *Aspergillus terreus* |
| *Aspergillus versicolor* | *Aspergillus versicolor* |
| *Aureobasidium pullulans* | *Aureobasidium pullulans* |
| *Blastomyces dermatitidis* | － |
| *Chaetomium globosum* | *Chaetomium globosum* |
| *Chaetomium funicola* | － |
| *Cladosporium cladosporioides* | *Cladosporium cladosporioides* |
| *Cordyceps javanica* | － |
| *Cunninghamella bertholletiae* | *Cunninghamella bertholletiae* |
| *Exophiala dermatitidis* | *Exophiala dermatitidis* |
| *Fusarium falciforme* | － |
| *Fusarium moniliforme* | *Fusarium verticillioides* |
| *Fusarium oxysporum* | *Fusarium oxysporum* |
| *Fusarium solani* | *Fusarium solani* |
| *Geotrichum candidum* | *Geotrichum* *candidum* |
| *Geotrichum capitatum* | － |
| *Lichtheimia corymbifera* | *Lichtheimia corymbifera* |
| *Malbranchea filamentosa* | － |
| *Mucor racemosus* | *Mucor racemosus* |
| *Paecilomyces variotii* | *Byssochlamys spectabilis* |
| *Penicillium brevicompactum* | *Penicillium brevicompactum* |
| *Penicillium chrysogenum* | *Penicillium chrysogenum* |
| *Penicillium corylophilum* | *Penicillium corylophilum* |
| *Purpureocillium lilacinum* | *Purpureocillium lilacinum* |
| *Pseudallescheria boydii* | *Scedosporium boydii* |
| *Rhizomucor pusillus* | *Rhizomucor pusillus* |
| *Rhizopus oryzae* | *Rhizopus oryzae* |
| *Rhizopus stolonifer* | *Rhizopus stolonifer* |
| *Sarocladium kiliense* | *Sarocladium kiliense* |
| *Sarocladium strictum* | *Sarocladium strictum* |
| *Scopulariopsis brevicaulis* | *Scopulariopsis brevicaulis* |
| *Scopulariopsis chartarum* | － |
| *Stachybotrys chartarum* | *Stachybotrys chartarum* |
| *Talaromyces marneffei* | － |
| *Trichoderma viride* | － |
| *Ulocladium consortiale* | － |

**Supplementary data 3**

Calmodulin and rDNA-ITS sequencing results of the 19 discrepant MALDI-TOF MS and BluePoint MoldID results for clinical isolates.

The “－” symbol indicates no matching species in calmodulin sequencing.

|  | **Calmodulin sequencing** | **rDNA-ITS** | **MALDI-TOF MS** | **BluePoint MoldID** |
| --- | --- | --- | --- | --- |
| **1** | *Aspergillus subramanianii* | *Aspergillus unguis* | *Aspergillus unguis* | Unidentified species |
| **2** | *Aspergillus fumigatus* | *Aspergillus fumigatus* | *Aspergillus fumigatus* | *Aspergillus flavus / oryzae* |
| **3** | *Aspergillus brunneoviolaceus* | *Aspergillus aculeatus* | *Aspergillus japonicus* | Unidentified species |
| **4** | *Aspergillus amoenus* | *Aspergillus versicolor* | *Aspergillus sydowii* | Unidentified species |
| **5** | *Aspergillus sydowii* | *Aspergillus sydowii* | *Aspergillus sydowii* | Unidentified species |
| **6** | *Aspergillus sydowii* | *Aspergillus caesiellus* | *Aspergillus sydowii* | *Aspergillus versicolor* |
| **7** | *Aspergillus subramanianii* | *Aspergillus sclerotiorum* | *Aspergillus sclerotiorum* | Unidentified species |
| **8** | *Aspergillus fumigatus* | *Aspergillus fumigatus* | *Aspergillus unguis* | Unidentified species |
| **9** | *Aspergillus fumigatus* | *Aspergillus fumigatus* | *Aspergillus sydowii* | Unidentified species |
| **10** | *Aspergillus flavus* | *Aspergillus caesiellus* | *Aspergillus sydowii* | *Aspergillus versicolor* |
| **11** | *Aspergillus fumigatus* | *Aspergillus fumigatus* | *Aspergillus fumigatus* | Unidentified species |
| **12** | *Aspergillus welwitschiae* | *Aspergillus sydowii* | *Aspergillus sydowii* | *Aspergillus versicolor* |
| **13** | *Aspergillus awamori* | *Aspergillus ochraceopetaliformis* | *Aspergillus pseudoglaucus* | Unidentified species |
| **14** | *Aspergillus flavus* | *Aspergillus unguis* | *Aspergillus unguis* | Unidentified species |
| **15** | *Aspergillus sydowii* | *Aspergillus sydowii* | *Aspergillus sydowii* | *Aspergillus versicolor* |
| **16** | *Aspergillus versicolor* | *Aspergillus versicolor* | *Aspergillus sydowii* | *Aspergillus versicolor* |
| **17** | － | *Aspergillus flocculosus* | Unidentified species | Unidentified species |
| **18** | *Aspergillus sydowii* | *Aspergillus flocculosus* | Unidentified species | Unidentified species |
| **19** | *Aspergillus sydowii* | *Aspergillus sydowii* | *Aspergillus sydowii* | *Aspergillus versicolor* |
